# Presentation and Verification of an Optimal Operating Scheme Aiming at Reducing the Ground Vibration Induced by High Dam Flood Discharge

**DOI:** 10.3390/ijerph17010377

**Published:** 2020-01-06

**Authors:** Jijian Lian, Lin Chen, Chao Liang, Fang Liu

**Affiliations:** 1 State Key Laboratory of Hydraulic Engineering Simulation and Safety, Tianjin University, Tianjin 300350, China; jjlian@tju.edu.cn (J.L.); xiaolinzi@tju.edu.cn (L.C.); fangliu@tju.edu.cn (F.L.); 2 School of Civil Engineering, Tianjin University, Tianjin 300350, China; 3 Postdoctoral Workstation, Yellow River Engineering Consulting Co., Ltd., Zhengzhou 450003, China; 4 Postdoctoral Research Station of Water Conservancy Engineering, Hohai University, Nanjing 210098, China

**Keywords:** ground vibration, high dam flood discharge, hydro-elastic experiment, vibration prediction, vibration reduction, optimal operation scheme, prototype verification

## Abstract

Ground and environmental vibrations induced by high dam flood discharge from the Xiangjiaba hydropower station (XHS) has significant adverse effects on nearby building safety and the physical and mental health of surrounding residents. As an effective approach to simulate the flow-induced vibration of hydraulic structures, the hydro-elastic experiment approach has been extensively applied and researched by Chinese scholars, but the relevant systematic research is rarely reported in international journals. Firstly, the hydraulic and structural dynamic similarity conditions that should be satisfied by the hydro-elastic model are briefly reviewed and derived. A hydro-elastic model of the XHS was further constructed using self-developed high-density rubber, and the vibration isolation system (including open trenches and flexible connects) was applied to avoid the external disturbances of pump operation, vehicle vibration and other experiments in the laboratory. Based on the data of model and prototype dynamic tests, a back propagation (BP) neural network was established to map the acceleration of the physical model to the ground in the prototype. In order to reduce the ground vibration, experiments were carried out to meticulously evaluate the ground vibration intensity under more than 600 working conditions, and the optimal operation scheme under different discharge volumes is presented here in detail. According to the prototype test data in 2013, 2014, and 2015, ground vibrations were significantly reduced by applying the presented optimal operation principle which indicates that the presented hydro-elastic approach and the vibration attenuation operation scheme were effective and feasible.

## 1. Introduction

Due to the need for a comprehensive utilization of water resources, many hydropower engineering projects with high dams and large reservoirs have been built or are currently under construction. The natural environment is inevitably disturbed and many environmental and ecological issues can result [1,2,3,4,5,6,7] if insufficient water resources planning, unreasonable operation schemes, and obsolete construction technology are applied. The vibration problems of hydraulic structures (e.g., a dam body [8,9], a hydraulic gate [10,11,12], a gate pier [13,14], a guide wall [15,16], and a plunge pool floor [17]) induced by high dam flood discharge are originally an engineering problem that has an adverse impact on structural safety and normal operation. However, ground vibrations induced by the flood discharge of several large hydropower engineering projects have recently been observed, and adverse effects on the nearby building safety and the physical and mental health of surrounding residents have been identified [18,19,20]. Due to the expansion of the affected area, the vibration problem induced by flood discharge changes from an engineering problem to an environmental problem, and this will cause conflicts between the surrounding residents and the power producer and even lead to mass incidents if this problem is not properly handled. One of the authors has experienced this vibration problem in the surrounding buildings of the Xiangjiaba hydropower station (XHS). It is disturbing that one can feel the weak vibration of the floor and that the unreinforced doors and windows emit loud vibration noises during the flood discharge. According to the prototype test data in 2013, 2014, and 2015, the ground vibration induced by the flood discharge of the XHS was significantly reduced by applying the optimal operation principles presented in this article. It is comforting that the conflicts between the surrounding residents and the power producer have been effectively resolved and that more mass incidents have been avoided by using comprehensive measures including applications of the optimal operation scheme, economic compensation, and housing renovation.

**Figure 1:**
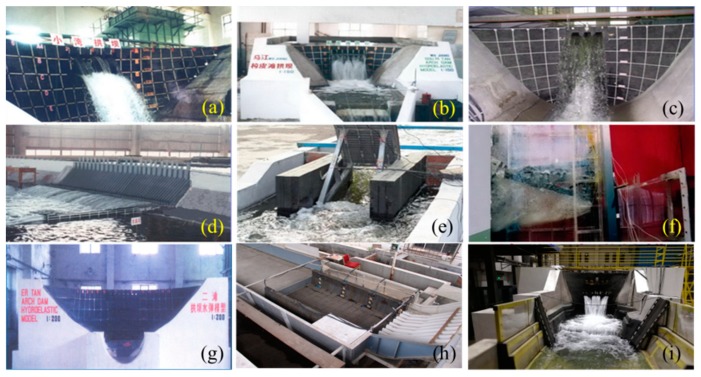
Engineering applications of the hydro-elastic experiments. (**a**) The arch dam of the Xiaowan hydropower project. (**b**) The arch dam of the Goupitan hydropower project. (**c**) The arch dam of the Laxiwa hydropower project. (**d**) The guide wall of the Three Gorges hydropower project. (**e**) The hydraulic gate of the Xinzheng hydropower project. (**f**) The hydraulic gate of the Three Gorges hydropower project. (**g**) The arch dam of the Ertan hydropower project. (**h**) The guide wall of the Xiangjiaba hydropower station (XHS). (**i**) The arch dam of Wudongde hydropower project.

Due to the extreme complexity of the hydraulic problem in practical engineering and the impossibility of a general solution for the N–S equation, hydraulic model testing satisfying a gravity similarity criterion is an effective and frequently used method. In order to obtain a structural vibration response induced by flood discharge excitation in engineering practice, Ettema et al. [21] suggest that the hydro-elastic model experiment can be applied, but the similarities for mass and stiffness of the structures in model and prototype are difficult to satisfy without using a specially customized material. In the 1980s, a hydro-elastic model experiment technique was presented by Cui et al. [22]. They used self-developed high-density rubbers instead of plexiglass to simulate the concrete for hydraulic structure construction. The density of self-developed rubber is equal to that of concrete, but the elastic modulus of rubber equals the concrete elastic modulus divided by the geometric scale, so the hydraulic and structural statics/dynamics similarity conditions between the real structure and the simulated model can be approximately satisfied [23]. This technique can reasonably simulate the flow-induced vibration of hydraulic structures, considering the complex situations in engineering practice in a relatively comprehensive way; thus, it has been applied to many hydropower projects, as shown in Figure 1. However, similar to the conventional hydraulic experiment, the flow aeration effect cannot be appropriately simulated in hydro-elastic experiments. Moreover, the similarities for the damping ratio and Poisson’s ratio cannot be satisfied [24,25,26]. According to the application and verification of the hydro-elastic experiments in engineering practice, it has been generally proved and widely acknowledged that the experimental accuracy is relatively high and acceptable.

It is worth pointing out that this hydro-elastic experiment technique has rarely been reported in international journals for unknown reasons, although this technology has been widely used to solve practical problems over the past 30 years and has been adequately reported in many Chinese academic journals [27,28,29,30,31,32]. In the present article, a detailed optimal operation scheme is presented based on an improved hydro-elastic experiment technique aiming at effectively reducing the ground vibration induced by the flood discharge of the XHS, and the prototype test results show that the presented operation scheme is reasonable and effective. The remainder of this article is organized as follows. Section 2 introduces basic information about the XHS and its ground vibration problem. In Section 3.1, the theoretical fundamentals of the hydro-elastic experiment are briefly reviewed and derived, and the improved hydro-elastic model for the dam, foundation, stilling basin, and reservoir of the XHS is then established. Moreover, the nonlinear mapping from the model vibration data to the prototype vibration data is determined and verified in Section 3.2. by applying the BP neural network prediction model. In Section 4, the optimal operation scheme for ground vibration reduction is presented based on a large number of model test data. The presented optimal scheme is then proved to be reasonable and effective according to the analysis of the prototype vibration data of recent years in Section 5. Moreover, the sources for the errors between the prediction and prototype test results are discussed, and the actual situation of the ground vibration under prototype working conditions is further described in Section 6. Finally, a conclusion is provided in Section 7.

## 2. The Ground Vibration Problem of the Xiangjiaba Hydropower Station (XHS)

**Figure 2:**
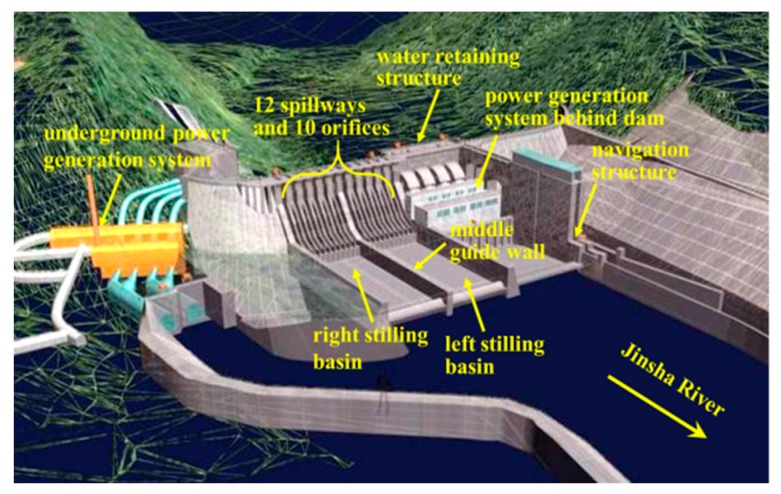
General layout of the Xiangjiaba hydropower station (XHS).

The XHS is the last cascade hydropower station in the lower reach of the Jinsha River. The main task of this engineering project is to generate electricity, improve navigation conditions, avoid flood hazard, and ensure irrigation water demand. Moreover, this project can also reduce sediment transport and reversely regulate the Xiluodu hydropower station in upstream reaches. The XHS controls a watershed area of 458,800 km^2^ which accounts for 97% of the whole Jinsha River basin area. The maximum height of the dam is 162 m, and the installed capacity of the XHS is 6400 MW. As shown in Figure 2, this hydropower engineering project is mainly composed of water retaining construction, flood and sand discharging construction, water transfer, and power generation systems behind the left part of the dam and under the ground of the right bank. There are 12 spillways and 10 orifices in the dam body of the XHS, and an orifice is arranged between each pair of spillways. Two symmetrical stilling basins are included in the XHS project, and a middle guide wall is constructed between the left and right stilling basins. The flow discharged from the left half spillways and orifices falls into the left stilling basin, and the flow discharged from the right half spillways and orifices falls into the right stilling basin. More detailed information on the hydraulic structures can be found in Section 3.1.3.

**Figure 3:**
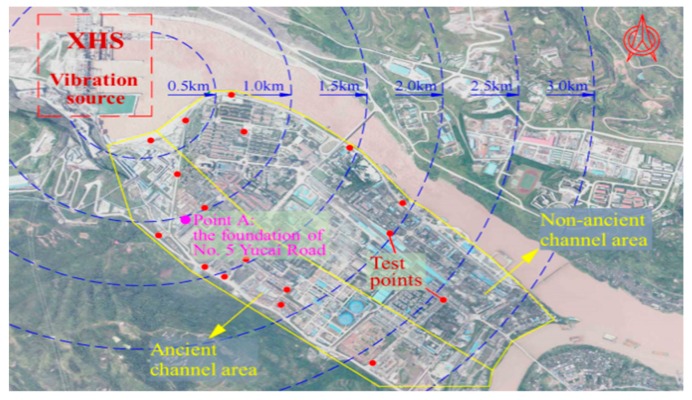
Surrounding ground of the Xiangjiaba hydropower station (XHS).

As shown in Figure 3, the XHS is approximately 1.5 km away from the urban area of Shuifu County, and some buildings are constructed on the ancient river channel area where the soil is composed of gravel overburden and is relatively soft. Therefore, the high water head of the dam, the close distance between the residential area and vibration source, and the soft soil ground jointly contribute to the vibration amplification effect of the surrounding ground of the XHS. On 10 October 2012, the reservoir impoundment of the XHS started, and the orifices in dam body began to discharge water on 12 October 2012. In the initial reservoir impoundment period of the XHS in 2012, the upstream water level was lower than the elevation of the crest of the spillways, so the water was discharged only from the orifices.

However, the surrounding ground vibration of the XHS was observed and caused strong oscillations in the doors and windows of the surrounding buildings during the flood discharging process. This vibration phenomenon and the vibration-induced noise led to fear and panic among the surrounding residents and had significantly adverse effects on the strength and safety of surrounding structures. The maximum ground acceleration reached 0.82 gal when the flood volume discharged from the dam body was only 10,000 m^3^/s (2 year flood), so it was concerning that the vibration would increase when the flood volume discharged from the dam body reached 34,800 m^3^/s (100 year flood) and even 48,400 m^3^/s (5000 year flood). Therefore, the ground vibration intensity needs to be effectively reduced. Mass incidents and even social unrest can result if this engineering problem is not handled properly.

## 3. Prediction of Prototype Vibrations Based on a Hydro-Elastic Experiment

### 3.1. The Hydro-Elastic Model

In order to appropriately simulate the fluid–solid coupling system and obtain accurate and undisturbed test data, the hydro-elastic model was constructed according to both the hydraulic and structural dynamic similarity conditions, which are briefly reviewed and deduced in subsequent sections. Moreover, the vibration sensor arrangement, the dynamic test system, and the vibration isolation system are elaborated in the following analysis.

#### 3.1.1. Similarity of Hydraulic Conditions

The similarity of hydraulic conditions is essentially the similarity between the flow-induced dynamic excitations in prototype and scale model tests. According to a series of prototype and model tests, it is widely believed that the wall pressure induced by flow separation and diffusion when the external boundary or flow condition sharply changes is mainly influenced by the motion of a large-scale eddy with low frequency. Under the condition of a high Reynolds number, the motion of a large-scale eddy can be well simulated, and the fluctuating pressure in a prototype can thus be calculated by the fluctuating pressure measured in the model test according to the gravity similarity criterion (i.e., the Froude similarity criterion). The dynamic load in the vibration problem of the XHS is mainly composed of fluctuating pressures in the energy dissipation zone of the stilling basin and is generated by a rapidly varied flow with strong separation and turbulent mixing. Therefore, the hydro-elastic model can be appropriately designed according to the gravity similarity criterion.

#### 3.1.2. Similarity of Structural Dynamic Conditions

The similarity of the structural dynamic condition is essentially the similarity between the dynamic behaviors of the structures in the model and prototype under flow-induced excitation. This similarity criterion is related to the structural frequencies, mode shapes, damping, etc. and includes the geometric, physical, motion, and boundary similarity conditions of the structure.

(1) Geometric Condition Similarity of the Structure

The geometric similarity condition will be satisfied when the experiment model is constructed by reducing the actual structure in equal proportion. Therefore, the following similarity scales can be obtained:

(1) $$\lambda_{S}=\lambda_{L}^{2};\;\lambda_{V}=\lambda_{L}^{3};\;\lambda_{\varepsilon }=1;\;\lambda_{\vartheta }=1;\;\lambda_{u}=\lambda_{L}$$

where $\lambda_{L}$, $\lambda_{S}$, $\lambda_{V}$, $\lambda_{\varepsilon }$, $\lambda_{\vartheta }$, and $\lambda_{u}$ are the length, area, volume, linear strain, angular strain, and linear displacement scales, respectively.

(2) Physical Condition Similarity of the Structure

The similarity of the physical condition requires that the mechanical properties of the structural materials and the stresses induced by external loads must be similar. It can be conveniently calculated that the following relationships should be satisfied according to the physical equations of elasticity under the condition of linear elastic deformation.

(2) $$\lambda_{\mu }=1;\;\lambda_{\sigma }=\lambda_{E}\cdot \lambda_{\varepsilon };\;\lambda_{\tau }=\lambda_{G}\cdot \lambda_{\vartheta }$$

where $\lambda_{\mu }$, $\lambda_{\sigma }$, $\lambda_{\tau }$, $\lambda_{E}$, and $\lambda_{G}$ are the Poisson’s ratio, normal stress, shear stress, elastic modulus, and shear modulus scales, respectively.

When the material density scale $\lambda_{\rho }=1$, the following relationship can be obtained:

(3) $$\lambda_{\sigma }=\lambda_{\tau }=\lambda_{E}=\lambda_{G}=\lambda_{\vartheta }.$$

(3) Movement Condition Similarity of the Structure

Generally, the dynamic equation of the structure subjected to random excitation can be expressed as follows:

(4) $$M\ddot{u}\left(t\right)+C\dot{u}\left(t\right)+Ku\left(t\right)=P\left(t\right)$$

where the matrices M, C, and K are the structural mass, damping, and stiffness matrices, respectively; P denotes the flow-induced fluctuating pressure; $\ddot{u}$, $\dot{u}$, and *u* represent the acceleration, velocity, and displacement of the structure; *t* denotes time. 

Considering the fluid–solid interaction effect, Equation (4) can be rewritten as:

(5) $$M_{s}\ddot{u}\left(t\right)+M_{w}\ddot{u}\left(t\right)+C\dot{u}\left(t\right)+ELZu\left(t\right)=P\left(t\right)$$

where the matrices $M_{s}$ and $M_{w}$ denote the mass matrices of the structure and the water, respectively; *E* and *L* denote the elastic modulus and length characteristics of the structure; Z represents the dimensionless constant matrix determined by the structural constraints. The sum of the matrices $M_{s}$ and $M_{w}$ is the total mass matrix M.

The following relationship can be deduced from Equation (5).

(6) $$\frac{\lambda_{Ms}\lambda_{\dot{u}}}{\lambda_{t}}=\frac{\lambda_{Mw}\lambda_{\dot{u}}}{\lambda_{t}}=\lambda_{c}\lambda_{\dot{u}}=\lambda_{E}\lambda_{L}\lambda_{u}=\lambda_{\bar{P}}\lambda_{L}^{2}=\lambda_{P}\lambda_{L}^{2}$$

where $\bar{P}$ denotes the flow-induced average pressure; the parameters $\lambda_{Ms}$, $\lambda_{Mw}$, $\lambda_{c}$, $\lambda_{E}$, $\lambda_{u}$, $\lambda_{\dot{u}}$, $\lambda_{\bar{P}}$, $\lambda_{P}$, and $\lambda_{t}$ represent the structural mass, water mass, damping, elastic modulus, structural displacement, structural velocity, average pressure, fluctuating pressure, and time scales, respectively.

Since the experiment model is designed according to the gravity similarity criterion, the following relationship can be obtained.

(7) $$\lambda_{t}=\lambda_{L}^{0.5},\;\lambda_{\bar{P}}=\lambda_{P}=\lambda_{L}.$$

Because of the geometric similarity of the structure, the following equation can be easily obtained.

(8) $$\lambda_{u}=\lambda_{L}.$$

Substituting Equations (7) and (8) into Equation (6) yields:

(9) $$\lambda_{Ms}=\lambda_{\gamma s}\cdot \lambda_{L}^{3}=\lambda_{Mw}=\lambda_{\gamma w}\cdot \lambda_{L}^{3}=\lambda_{C}\lambda_{\dot{u}}=\lambda_{E}\lambda_{L}^{2}=\lambda_{L}^{3}$$

where $\lambda_{\gamma s}$ and $\lambda_{\gamma w}$ denote the structural bulk density and liquid bulk density scales, respectively.

Considering that the liquid bulk densities (i.e., the water densities) in the prototype and physical model are the same, the following equation can be given.

(10) $$\lambda_{\gamma s}=1,\;\lambda_{C}=\lambda_{L}^{2.5},\;\lambda_{E}=\lambda_{L},\;\lambda_{\xi }=1$$

where $\lambda_{\xi }$ represents the damping ratio scale, respectively.

In Equation (10), the bulk density, damping coefficient, and elastic modulus scales for the physical model are determined. 

(4) Boundary Condition Similarity of the Structure

In order to satisfy the boundary condition similarity, the boundary constraints in the physical model should be as close as possible to those used in practical engineering. For the design of the hydro-elastic model for the XHS, the key point is the selection of a foundation simulation range. Based on a study of foundation boundary determination of an arch dam in a hydro-elastic experiment [33], a sufficiently large foundation (500 m long, 400 m wide, and 90 m deep in the prototype) is considered in the hydro-elastic model.

#### 3.1.3. Model Construction, Vibration Sensor Arrangement, and Dynamic Test System

**Figure 4:**
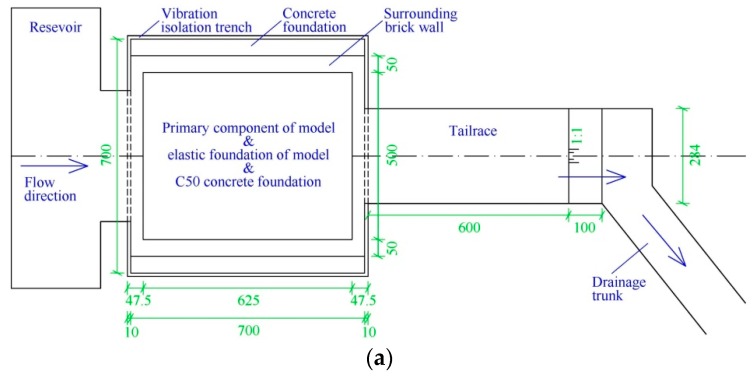

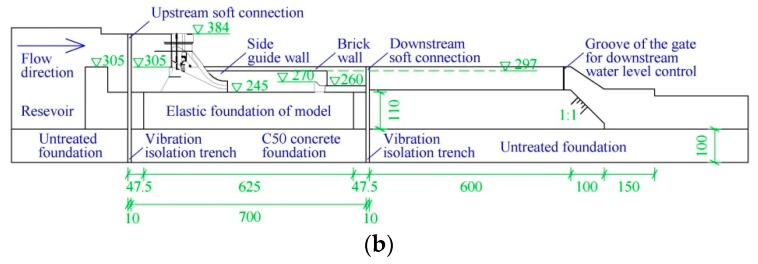
General arrangement of the hydro-elastic model. (**a**) Top-view image. (**b**) Profile map.

Comprehensively considering the scale effect and the economy cost, the geometric scale for the hydro-elastic model (denoted by *λ_L_*) is set to 1:80. The spillway dam section, two stilling basins, and the foundation (6.25 m long, 2 m wide, and 1.13 m deep in the physical model) are included in the hydro-elastic model. In the experiments, the flow rate is measured using the rectangular weir, and the water levels of the upstream and downstream are measured using the needle water level gauge. Figure 4 illustrates the general arrangement of the hydro-elastic model, in which the key parts of the model and their dimensions are described in detail.

**Figure 5:**
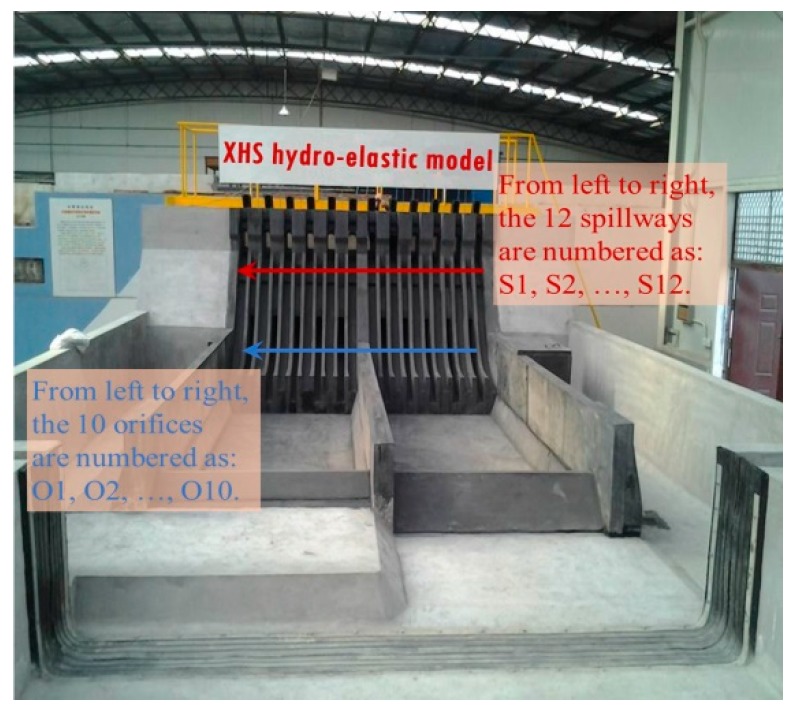
Photograph of the hydro-elastic model.

**Figure 6:**
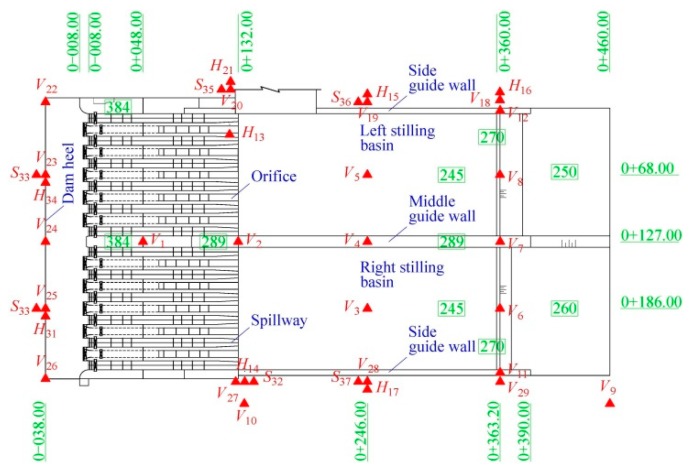
Acceleration sensor arrangement.

In Figure 5 and Figure 6, a photograph of the hydro-elastic model and the acceleration sensor arrangement are shown, respectively. According to the above analysis, the hydro-elastic model should be designed on the basis of the gravity similarity law and constructed with material with a high density ($\lambda_{\gamma s}=1$) and a low elastic modulus ($\lambda_{E}=\lambda_{L}$), and its damping ratio and Poisson’s ratio should be equal to the construction material (concrete) used in engineering practice ($\lambda_{\xi }=1$, $\lambda_{\mu }=1$). It can be seen in Figure 5 that the hydro-elastic model is made of a special material that is a kind of high-density rubber developed by the authors’ research team. This high-density rubber satisfies the aforementioned density scale and elastic modulus scale, but it is quite difficult to control the damping ratio and Poisson’s ratio of the self-developed material in the manufacturing process. Due to the applications in many hydropower engineering projects and the corresponding prototype verifications, there are good reasons to believe that the errors induced by the dissimilarities of the material damping ratio and Poisson’s ratio will not have a significant impact on the dynamic test results. The discussion for the effect of Poisson’s ratio dissimilarity on the hydro-elastic experiment results is given in detail in Section 3.2.

**Figure 7:**
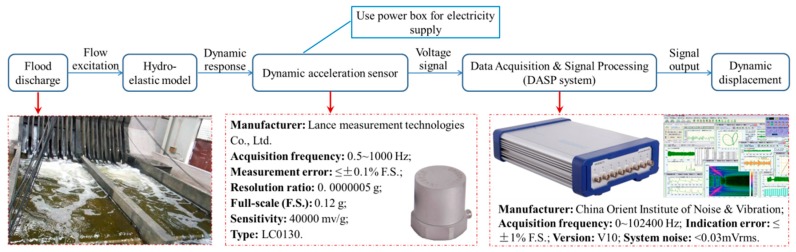
Dynamic testing system.

In Figure 6, the capital letter *V* represents the vertical acceleration sensor, and the capital letters *H* and *S* represent the horizontal sensors that are perpendicular and parallel to the direction of flow, respectively. In total, 37 acceleration sensors are arranged in the hydro-elastic model, including 24 vertical acceleration sensors, 6 horizontal sensors that are perpendicular to the flow direction, and 7 horizontal sensors that are parallel to the flow direction. The piezoelectric acceleration sensor produced by Lance Test Technology Co., Ltd., headquartered in Ohio, USA, was applied to measure the acceleration, and the INV data acquisition system produced by China Orient Institute of Noise & Vibration was used for signal acquisition and processing. In Figure 7, the technical parameters for the acceleration sensor and data acquisition system in the dynamic testing system are illustrated.

#### 3.1.4. Ground Vibration Isolation System

**Figure 8:**
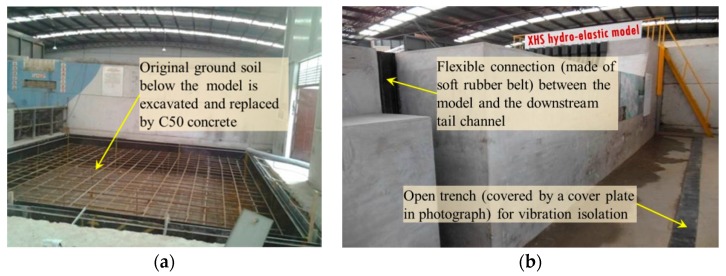
Vibration isolation system. (**a**) Excavation and replacement of the original ground soil. (**b**) Flexible connection and vibration isolation trench.

In order to avoid the interferences of background noise, pump operation, vehicle vibration, and other experiments in the laboratory with respect to the acceleration signal, the vibration isolation system was designed and was applied in this hydro-elastic model. The original soil below the XHS model was excavated and replaced by C50 concrete, and the concrete foundation was 7 m long, 7 m wide, and 1.5 m deep. The elastic foundation made of high-density rubber was then constructed above this concrete foundation (shown in Figure 4b). Moreover, four open trenches (0.1 m wide and 1.5 m deep) were arranged around the concrete foundation to cut off the lateral vibration transmission path. In addition, flexible connections made of a relatively soft water-tight rubber belt were used to connect the hydro-elastic model with the upstream reservoir, as well as the hydro-elastic model with the downstream tailrace. In this case, the hydro-elastic model was constructed on a rigid concrete foundation, and the vibration propagation paths above and below ground were cut off by the flexible connections and open trenches, respectively. Thus, the interferences of external vibration and noise could be effectively eliminated. In Figure 8, the vibration isolation system is illustrated.

### 3.2. The Effect of Poisson’s Ratio Dissimilarity on the Hydro-Elastic Experiment Results

Due to the well-developed similarity theory, the hydraulic model experiment is widely used to estimate hydraulic parameters and improve hydraulic conditions before any practical engineering is completed. As many hydropower engineering projects with high water head, high flow velocity, and enormous discharge energy have been built or are currently under construction, many researchers have tried to extend the model testing technique to the vibration problem induced by flood discharge. In the 1960s, the original hydro-elastic experiment technique was presented to only simulate the elastic behavior of a suspender on a hydraulic gate. The abnormal hydro-elastic experiment method [34] was then presented, and stiffness and mass similarity conditions were satisfied by thickening the structure components and adding lead blocks to the structure surfaces, respectively. However, this method has many disadvantages, such as the difficult construction of the experiment model, low experimental accuracy, and the impossibility of simultaneous simulations for bending and torsional vibrations [34]. The invention of high-density rubber, which can satisfy the density and elastic modulus similarity conditions very well, resulted in great progress in the experiment research of hydraulic structure vibrations induced by flood discharge. Although the Poisson’s ratio of high-density rubber is reduced to 0.35–0.4, which is obviously lower than that of ordinary rubber [35], it is still obviously higher than that of reinforced concrete. Generally, the Poisson’s ratios of plain concrete and reinforcing bars are 0.167 and 0.3, respectively, and the Poisson’s ratio of reinforced concrete is considered to be 0.2 in the Code for Design of Concrete Structures (GB50010-2010).

In order to further analyze the accuracy and generalizability of the hydro-elastic experiment technology, the effects of Poisson’s ratio on the dynamic characteristics of hydraulic structures were subsequently investigated in different situations.

For the simplest situation, in order to test the elastic modulus of high-density rubber material, a cantilever beam of high-density rubber was produced, and an acceleration sensor was installed on the free end of this cantilever beam. The cantilever beam was then struck slightly by a hammer, and the periodic acceleration history with decreasing amplitude was obtained. The fundamental natural frequency of a cantilever beam is equal to the reciprocal of an acceleration period and can also be calculated by the following formula according to Euler–Bernoulli theory.

(11) $$f=\frac{1.76}{\pi l^{2}}\sqrt{\frac{EI}{\rho S}}$$

where *f*, *l*, *E*, *I*, *ρ*, and *S* denote the fundamental frequency, length, elastic modulus, moment of inertia of cross sections, density, and cross section area, respectively. Considering the natural frequency that is already known, the elastic modulus *E* is the only unknown variable included in the above equation. Therefore, the elastic modulus of high-density rubber can be easily obtained. Poisson’s ratio is not involved in Equation (11), which is frequently used to test the elastic modulus of different materials. Moreover, Poisson’s ratio can be ignored in the whole process of modal analysis for beams according to Euler–Bernoulli theory. This indicates that at least in this special situation, Poisson’s ratio has no effect on the structure dynamic characteristics.

Considering the more complex situation, the basic dynamic equation for the multi degree of freedom (MDOF) system can be given as follows.

(12) $$M\ddot{u}+C\dot{u}+Ku=P\left(t\right)$$

where *M*, *C*, and *K* denote mass, damping, and stiffness matrices of this MDOF system, respectively; $\ddot{u}$, $\dot{u}$, and *u* denote structural acceleration, velocity, and displacement, respectively; *P* denotes the external excitation; *t* represents time. Different Poisson’s ratios will lead to different stiffness matrices, which is the main cause of the errors in dynamic responses. The plane structure is taken into consideration to further investigate how much error can be generated by differences in Poisson’s ratios. Without a loss of generality, this plane structure is considered to be divided by rectangular grids. The stiffness matrix of the rectangular element $K^{\left(e\right)}$ can then be calculated. We divide each element in the stiffness matrix with a 0.2 Poisson’s ratio (denoted as ${K^{\left(e\right)}|}_{\mu =0.2}$) by the corresponding element in the stiffness matrix with a 0.4 Poisson’s ratio (denoted as ${K^{\left(e\right)}|}_{\mu =0.4}$), and the following result can be obtained:

(13) $$\begin{array}{cc} \left({K^{\left(e\right)}|}_{\mu =0.2}\right)\cdot & \frac{}{\left({K^{\left(e\right)}|}_{\mu =0.4}\right)}\\ & =\left[\begin{array}{cccccccc} 1.07 & 0.86 & 0.94 & -2 & 1.07 & 0.86 & 0.5 & -2\\ 0.86 & 1.07 & -2 & 0.5 & 0.86 & 1.07 & -2 & 0.94\\ 0.94 & -2 & 1.07 & 0.86 & 0.5 & -2 & 1.07 & 0.86\\ -2 & 0.5 & 0.86 & 1.07 & -2 & 0.94 & 0.86 & 1.07\\ 1.07 & 0.86 & 0.5 & -2 & 1.07 & 0.86 & 0.94 & -2\\ 0.86 & 1.07 & -2 & 0.94 & 0.86 & 1.07 & -2 & 0.5\\ 0.5 & -2 & 1.07 & 0.86 & 0.94 & -2 & 1.07 & 0.86\\ -2 & 0.94 & 0.86 & 1.07 & -2 & 0.5 & 0.86 & 1.07 \end{array}\right] \end{array}.$$

The 62.5% elements in the above constant matrix are very close to 1, which means that the stiffness effect generated by the material with a 0.2 Poisson’s ratio is approximately equivalent to that generated by the material with a 0.4 Poisson’s ratio. Some matrix elements also show relatively large differences from 1, which indicates that the stiffness effects generated by the materials with different Poisson’s ratios are quite different. These non-equivalent stiffness effects are illustrated in the following figure.

**Figure 9:**
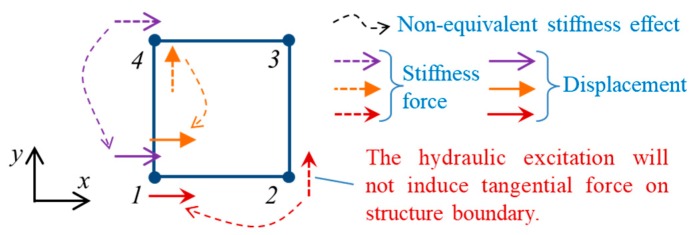
Non-equivalent stiffness effects generated by the materials with 0.2 and 0.4 Poisson’s ratios.

As shown in Figure 9, there are three non-equivalent stiffness effects acting on each node in a rectangular element. It is worth pointing out that the hydraulic excitation will not directly induce tangential force on the structure boundary and that all tangential forces are generated by tangential deformations that are caused by normal forces. Therefore, it can be reasonably inferred that the tangential forces are generally smaller than the normal forces, which will reduce two of three non-equivalent stiffness effects shown in Figure 9. According to the above analysis, it can be concluded that the influence of Poisson’s ratio variation (from 0.2 to 0.4) on the dynamic characteristics of the plane structure is very limited due to the similar stiffness and Rayleigh damping matrices. This only provides a possible way to explain and understand why the Poisson’s ratio dissimilarity will not generate significant effects on the structural vibration mode and dynamic behavior. A more reliable quantitative analysis of the actual hydraulic structure should be given to further discuss the possible error induced by hydro-elastic experiments. Therefore, a comparative analysis for the natural vibration modes of actual hydraulic buildings with different Poisson’s ratios was carried out on the basis of numerical simulation.

**Figure 10:**
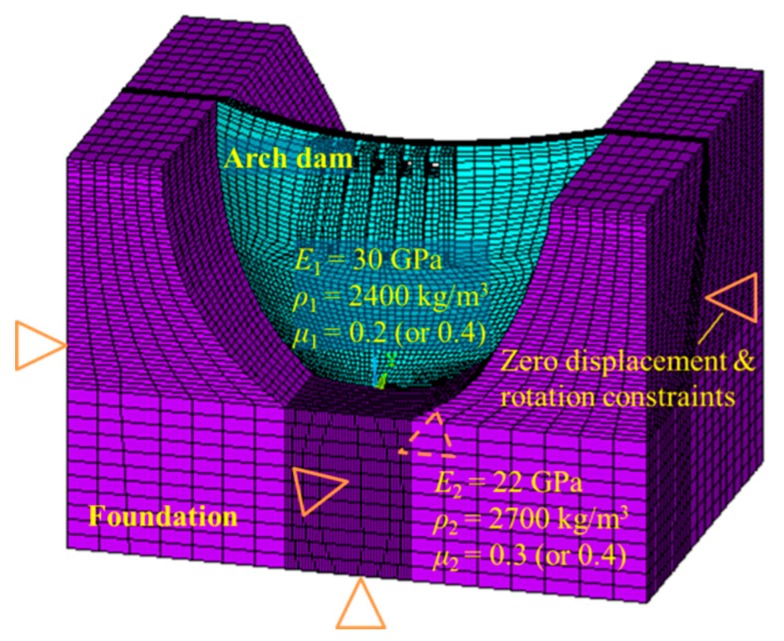
Numerical model for practical engineering.

The numerical model of the XHS and the surrounding ground could not be established due to the limited information. Thus, the numerical model of another hydraulic building was taken as an example to analyze the effect of Poisson’s ratio on the structural dynamic characteristics. In Figure 10, the numerical model is illustrated, the related parameters are given, and the constraint condition is described. The variables *E*, *ρ*, and *μ* denote the elastic modulus, density, and Poisson’s ratio, respectively, and Subscripts 1 and 2 relate to the associated variables of the dam body and foundation, respectively. In order to focus on the vibration mode of the dam body, zero displacement and rotation constraints were applied to the front, back, left, right, and bottom surfaces of the foundation.

In Table 1, a comparison between the natural frequencies of the numerical model with different Poisson’s ratios is given. The natural frequencies for the actual material model and the high-density rubber model are very close to each other, and most of the frequency errors are less than 1%. The frequency error of the 3rd mode is the maximum error in the first 10 modes, which reaches 5.31%.

**Figure 11:**
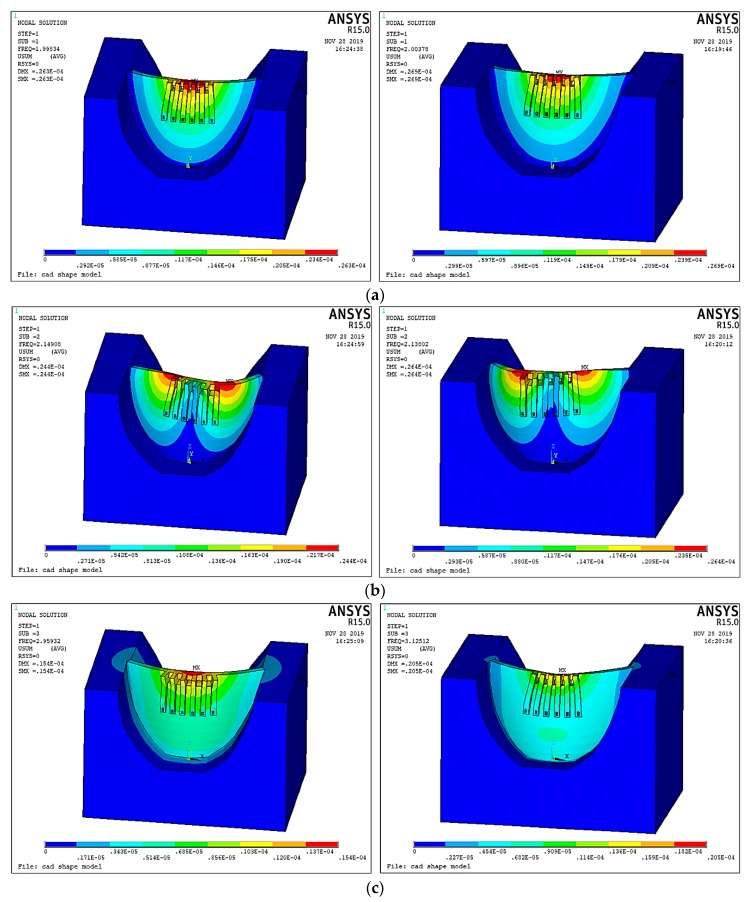
Comparison between the mode shapes calculated by the numerical models with different Poisson’s ratios. (**a**) 1st mode. (**b**) 2nd mode. (**c**) 3rd mode.

The mode shape is also important information in modal analysis and has a significant influence on structural dynamic behavior. Therefore, a comparison between the mode shapes calculated by the numerical models with different Poisson’s ratios is given in the following figure. As shown in Figure 11, the two mode shapes for the first three vibration modes corresponding to different Poisson’s ratios are very similar to each other. For the vibration modes of higher orders, the mode shapes corresponding to different Poisson’s ratios are generally similar, but the local differences are increased. As the flow excitation frequency is very low (usually lower than 1 Hz), the contribution of a low-order mode to the hydraulic structural vibration is much greater than that of a high-order mode. Therefore, the local differences between the high-order mode shapes corresponding to different Poisson’s ratios will not induce significant error in the dynamic behavior.

According to the above analysis, the first three natural frequencies and mode shapes corresponding to Poisson’s ratios of the actual material and the high-density rubber are almost equal to each other. This provides strong evidence for the conclusion that Poisson’s ratio has very limited influences on structural dynamic characteristics and the errors caused by the dissimilarity of Poisson’s ratio can be ignored in engineering practice. Therefore, it is believed that the hydro-elastic experiment technology is accurate and reliable, and can be applied to analysis of hydraulic structure vibrations induced by flood discharge in other engineering projects.

The damping ratios respectively calculated by different numerical models are not given. Due to the complex spatial structure, water-stop rubber damping, and the radiation damping effect generated by fluid–structure coupling, the damping condition of the hydraulic structure is extremely complicated. This complicated damping condition cannot be accurately simulated in numerical analysis regardless of Poisson’s ratio changes, so a detailed comparison and analysis of the damping of numerical models with different Poisson’s ratios are not provided in this paper. According to the above analysis, the damping conditions corresponding to different Poisson’s ratios will be similar if the Rayleigh damping is applied due to the same mass matrix and the similar stiffness matrix.

Although the above modal analysis is based on a specific engineering project, the conclusion that the Poisson’s ratio has very limited influence on the dynamic response of the hydraulic structure is believed to be generally applicable. The combined method of a hydro-elastic experiment, a prototype test, and numerical simulation has already been applied to many hydropower projects, and some reliable and beneficial results have been obtained. In Table 2 and Table 3, the research on the vibration modes and dynamic responses of hydraulic structures using hydro-elastic experiment technology in existing references are summarized, respectively.

All the references included in Table 2 and Table 3 are published in Chinese academic journals. As a developing country, the construction period of hydropower projects in China is relatively late, and due to the natural environment, the project scale is generally large. Thus, there is a more important requirement in China to further study the vibration safety issues induced by the high dam flood discharge. Comprehensively considering various research methods, the hydro-elastic experiment technology is the most feasible and accurate method with a relatively solid theoretical basis. According to the aforementioned analysis and the existing references, the dissimilarity of Poisson’s ratios will not cause significant errors in the hydro-elastic experiment, and this technology is effective and generally applicable to the analysis of hydraulic structure vibrations induced by flood discharge.

### 3.3. Vibration Prediction Model

#### 3.3.1. Back-Propagation Neural Network Prediction Model

It is known that the back-propagation (BP) neural network prediction model is an effective and frequently used feedforward network with strong nonlinear mapping ability. Therefore, we used a BP neural network to establish the nonlinear mapping between the vibration accelerations measured in the hydro-elastic experiments and the prototype tests.

Several recent studies have been focused on the spatial distribution of the ground vibration intensity under various working conditions. Detailed analyses of vibration spatial distribution under different working conditions have been provided by Lian et al. [18] and Liang et al. [19]. The vibration intensity of point *A* is obviously greater than that of other test points of the surrounding ground. Based on large-scale numerical analysis considering the unlimited surrounding ground by an infinite element technique, a general propagation law of ground vibration under various working conditions was obtained [52]. Briefly speaking, the propagation law shows obvious vibration amplification effects in the area close to the vibration source (i.e., the XHS) and the ancient river channel region, which is consistent with the results of comprehensive prototype observations made by Luo et al. [53]. Point *A* is located on the side near the vibration source in the ancient river channel area. Therefore, the vibration intensity of point *A* is greater than that of most other points in different working conditions, and the vibration level of the whole near-field will decrease if the vibration intensity of point *A* is reduced by the applying optimal operation scheme. Moreover, the prototype test data of this point are relatively adequate. Thus, the vibration of point *A* was selected as the analysis object in this study, and the BP neural network was trained to predict the vertical acceleration root mean square (RMS) of point *A*.

The input layer consists of 19 neurons, which represent the RMSs of the acceleration histories measured by 19 vertical acceleration sensors in the hydro-elastic model (the acceleration signals measured by the other 5 vertical acceleration sensors are unavailable). Moreover, the number of neurons in the hidden layer can be determined on the basis of the Kolmogorov theorem. The approximate relationship between the neuron number in the hidden layer *n*_2_ and the neuron number in the input layer *n*_1_ can be expressed as

(14) $$n_{2}=2n_{1}+1.$$

**Figure 12:**
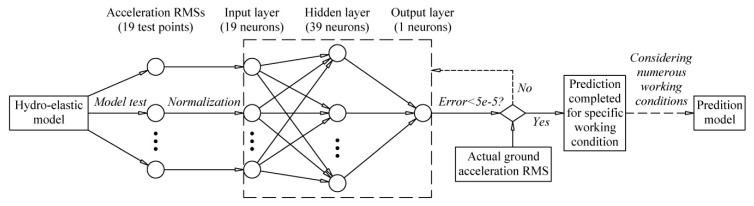
The establishment process of the prediction model.

Therefore, the number of hidden layer neurons was set as 39, and the standard BP neural network model with a network structure of 19-39-1 was established. The tan-sigmoid and linear transfer functions were used in the hidden and output layers, respectively. Moreover, the gradient descent learning algorithm with variable learning rate was applied in the model training, and the data normalization function in the MATLAB neural network toolbox (i.e., function *mapminmax*) was applied to normalize the input data, which significantly varied. The establishment of the BP neural network and the prediction calculation were mainly based on the MATLAB neural network toolbox, which was convenient. Figure 12 is shown to describe the establishment process of the prediction model more clearly. As research on BP neural networks has been published, the corresponding theoretical basis is not included in this article. 

#### 3.3.2. Comparison between the Results of the Prediction and Prototype Tests

**Figure 13:**
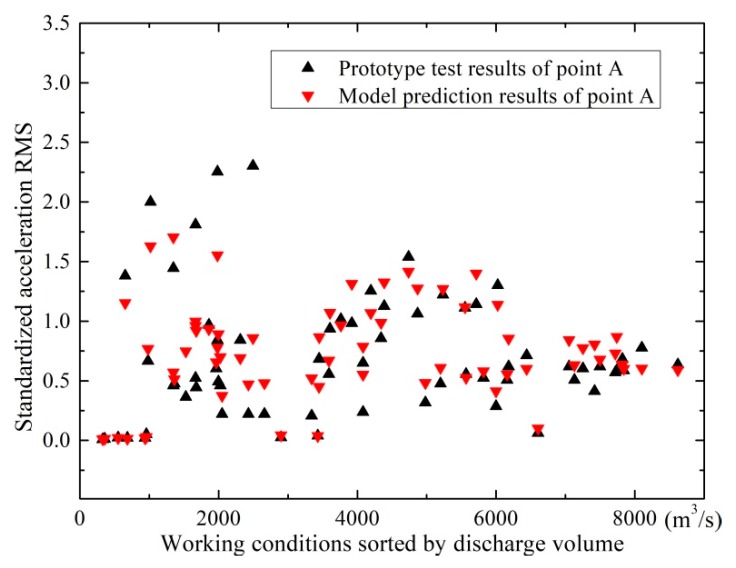
Comparison between the predicted and measured acceleration RMSs of point *A* under different working conditions.

In order to obtain a relatively accurate prediction model with good generalization ability, a large number of data, including the acceleration RMSs of the 19 test points on the hydro-elastic model (input data) and the acceleration RMSs of point *A* in the prototype (output data), under various working conditions, should be used to train the prediction model. Therefore, more than 150 working conditions of the XHS in the flood seasons of 2013 were reproduced in the hydro-elastic experiments, and the prototype and experimental results under these working conditions were applied in the model training. The trained model was then employed to predict the vertical acceleration RMSs of point *A* on the basis of experiment results under several critical working conditions in 2013 as well as many working conditions in 2014. In order to conveniently compare the results of the model prediction and prototype test, the acceleration RMSs involved in the following analysis are standardized by the baseline value, which is equal to 0.10 gal. In Figure 13, the prediction results are illustrated and compared with the prototype test results. It can be seen that the prediction values under most working conditions are in relatively good agreement with the prototype test results. For working conditions with low prediction accuracy, errors may come from environmental noise in the prototype, prototype and model test errors, and the calculation error of the BP neuron network model. The external disturbance in the prototype test is considered to significantly contribute to the error, because the measuring point *A* was arranged in a residential area where multiple interference sources exist. Thus, the disturbances of vehicle vibration, human activities, and other external noise make the prototype test results different from the prediction values. On the whole, it is considered that the predicted acceleration RMSs of point *A* are acceptable in engineering practice.

## 4. Optimal Operation Scheme Aiming at Ground Vibration Reduction

### 4.1. Basic Variation Law of Ground Vibration under Different Working Conditions

In order to investigate the basic variation law of ground vibration intensity under different working conditions, the effects of the spillway gate opening, the orifice gate opening, the upstream water level, and the downstream water level on the ground vibration intensity were studied. Although numerous experiments were carried out, only the results that show relative obvious regularity and can provide useful guidance to the presentation of an optimal operation scheme are described in the following analysis.

**Figure 14:**
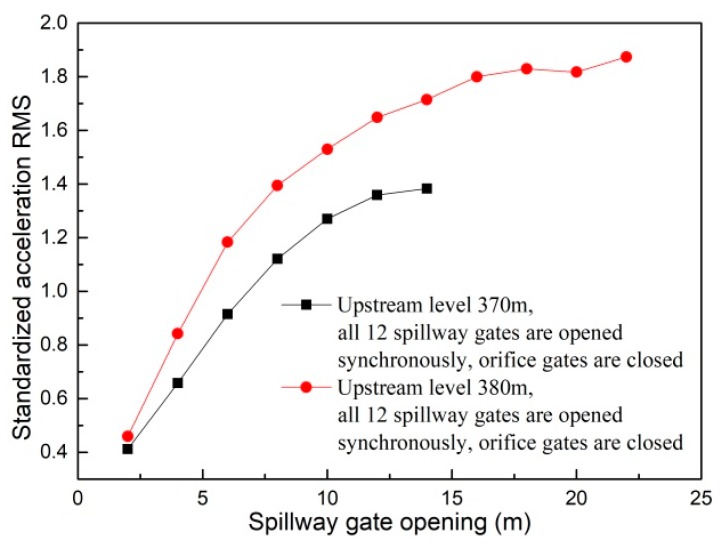
Vibration intensity of point *A* under working conditions with different spillway gate openings.

**Figure 15:**
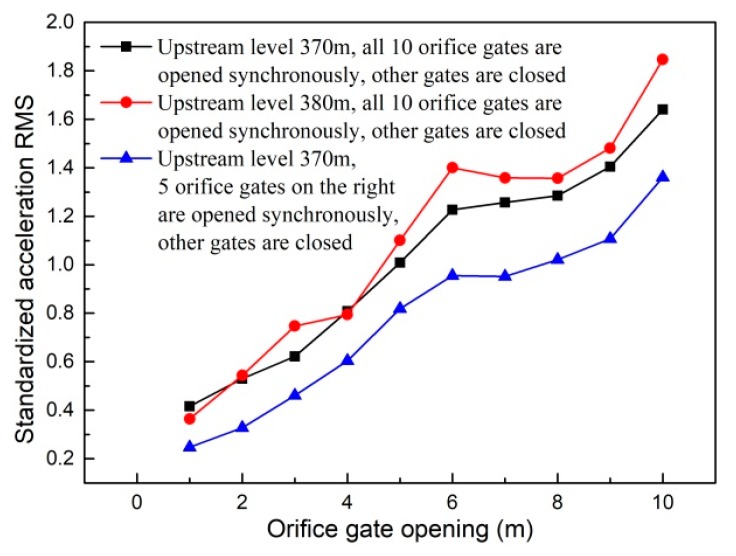
Vibration intensity of point *A* under working conditions with different orifice gate openings.

**Figure 16:**
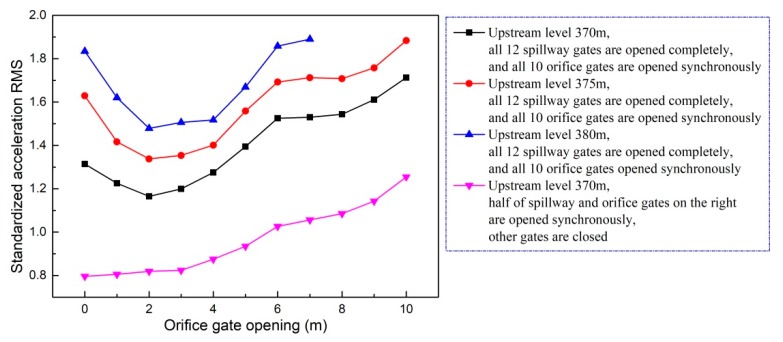
Vibration intensity of point *A* under working conditions with different orifice gate openings when the spillway gates are fully open.

Generally speaking, the ground vibration intensity increases as discharge volume and gate opening increase. As shown in Figure 14, when the flood is discharged only from the spillway, the increment of ground vibration intensity decreases as the gate opening increases. It can be seen in Figure 15 that the vibration intensity is significantly amplified when the flood is discharged only from orifices, and the orifice gate openings range from 5 to 7 m. This indicates that the specific orifice gate opening (ranging from 5 to 7 m) can induce more intense vibration and should be excluded from the optimal operation scheme aiming at vibration reduction. Moreover, Figure 16 shows the effect of the adverse orifice gate opening on the ground vibration intensity when the spillway gates are also open. The vibration amplification effect still exists when the spillway gate is fully open and the orifice gate is 5–7 m open. The hydraulic experiment results show that the flow-induced fluctuating pressures acting on the surface of the orifice and the floor of the stilling basin reach a maximum when the orifice gate opening is about 6 m open under different working conditions [54]. To focus on the subject of this article, the amplification effects of the specific opening range (5–7 m) of the orifice gate on the fluctuating pressure and ground vibration were not further analyzed. Based on Figure 16, it is clear that the vibration intensity will decrease if the orifice gate is approximately 2–3 m open under the condition of a fully open spillway gate. This implies that the collision and interaction between the flows discharged from spillways and orifices can induce energy dissipation; thus, the vibration energy transferred to the surrounding ground will be reduced, although the discharge volume will be increased. Moreover, a general conclusion can be drawn that the joint flood discharges of spillways with different openings and 2–3 m open orifices induce lower vibration intensity than another discharge mode under the condition of a similar flow rate. Based on the aforementioned basic rules, numerous experiments (more than 600 working conditions in total) were carried out, and a detailed optimal operation scheme is presented in the subsequent section.

### 4.2. Optimal Operation Scheme

**Figure 17:**
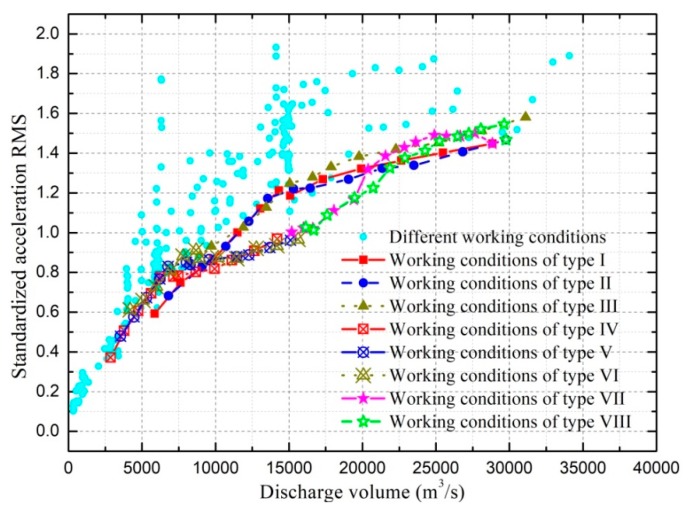
Comprehensive comparison of the predicted vibration intensities of point *A* under different working conditions.

In order to effectively reduce the ground vibration, a number of hydro-elastic experiments were carried out, and the corresponding acceleration RMSs of point *A* were predicted and standardized by dividing the reference value (i.e., 0.10 gal). In Figure 17, all of the prediction results under different working cases are illustrated, and the results with almost the lowest vibration intensities under the conditions of different discharges are highlighted. Due to space limitations, the specific operation parameters of the different working cases included in Figure 17 are given in Appendix A.

It can be seen in Figure 17 that the vibration intensities under the specific working conditions (where the spillway gates are fully open and the orifices are 2–3 m open) are significantly lower than the vibration intensities under most other working conditions with the same discharge volume. In the discharge range from 10,000 to 15,000 m^3^/s, the vibration intensities of point *A* are significantly reduced when only the right stilling basin is put into operation and the flow is not discharged from the left half spillway and orifice gates. As the discharge volume increases from 15,000 to 20,000 m^3^/s, it can be seen in Figure 17 that the ground vibration intensity will be smaller if the following operation scheme is applied. The right half spillway and orifice gates are fully and 2 m (or 3 m) open, respectively, the orifice gates corresponding to the left stilling basin are kept to be 2 m (or 3 m) open, and the spillway gate openings corresponding to the left stilling basin are gradually increased as discharge increases. Comprehensively considering the analysis results shown in Figure 17 and the discharge capacities for the individual spillway and orifice, the optimal operation scheme aiming at ground vibration reduction under different discharge conditions can be given as follows.

In the hydro-elastic experiment, ground vibrations under working conditions with 370 and 380 m upstream levels were mainly analyzed. The reason for this is that these two upstream levels are the dead storage level and the normal storage level, respectively, and the upstream levels of almost all the actual working conditions vary in the range from 370 to 380 m. According to the experiment results, similar effects on the ground vibration were generated by changing the gate opening mode in the same way regardless of the upstream water level. Moreover, two schemes of the working condition variation are considered in Figure 17: One scheme is to change the gate opening mode while the upstream level is fixed, and the other scheme is to change the upstream level while the gate opening mode is fixed. The working conditions with upstream levels of 372, 374, 376, and 378 m are also considered in both Figure 17 and the optimal operation scheme. Therefore, it is considered that the optimal operation scheme proposed in Table 4 is applicable to conditions with different upstream levels. 

## 5. Prototype Verification

**Figure 18:**
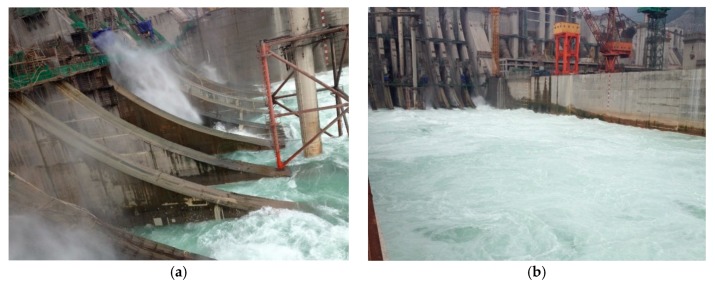
Photographs of the flow discharge operation of the XHS. (**a**) Flow pattern of orifice flow. (**b**) Flow pattern in plunge pool.

In order to verify the effectiveness of the aforementioned research results, the valuable data obtained by the prototype dynamic tests under different working conditions were analyzed and compared. Figure 18 provides photographs of the flow discharge operation of the XHS.

**Figure 19:**
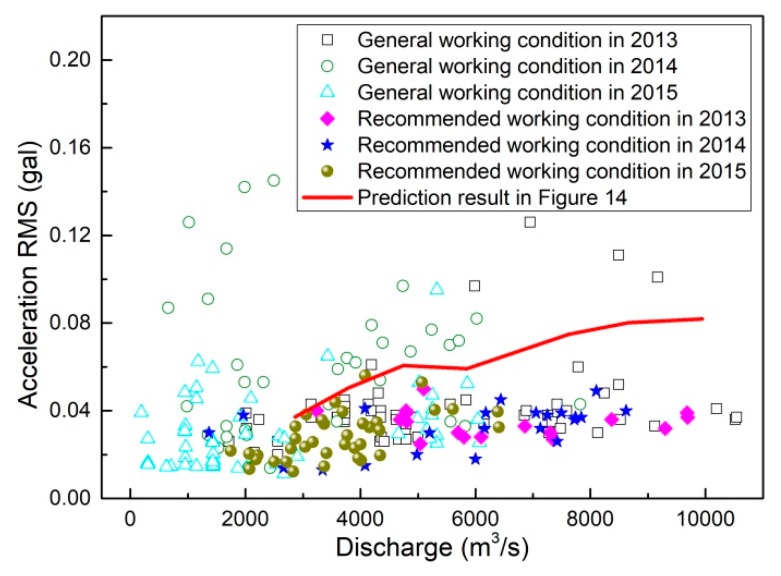
Comparison between the prediction and prototype test results.

The relatively high ground vibration intensity under relatively small discharge working conditions in 2012 was due to the fact that the flow was discharged only from orifices and the spillways were not put into operation. Following the suggestions of an optimal operation scheme (presented in Section 4) that was submitted to the management department of the XHS in the flood season of 2013, the ground vibration intensity was significantly reduced by the simultaneous discharge of the spillways and orifices. Detailed information is given in Table 5. In order to illustrate the effectiveness of the presented optimal operation scheme in detail, almost all of the working conditions and the corresponding ground vibration intensities in 2013, 2014, and 2015 are given in Figure 19.

As shown in Figure 19, the ground vibration intensities under the recommended working conditions in Section 4 are almost the lowest of the vibration intensities under all working conditions, which indicates that the ground vibration can be effectively reduced by applying the optimal operation scheme. It is clear from Figure 19 that the prediction approach combining hydro-elastic experiments and BP neural network calculations provides conservative results, especially for the discharge volume ranging from 6000 to 10,000 m^3^/s. Although there are obvious deviations between the predicted acceleration RMSs and the prototype test results, the vibration variation law under different working conditions and the optimal operation principle for vibration reductions are consistent with the actual situation to a great extent, which is of great significance in solving the ground vibration problem. This implies that the prediction approach for ground vibration is essentially reasonable and effective, but some improvements and correction methods should be further developed to improve the accuracies of the hydro-elastic experiment and the BP neural network calculation. Due to the small upstream inflow, the maximum discharge volume in the flood seasons of 2013, 2014, and 2015 was about 10,540 m^3^/s; thus, the ground vibration intensity and the fitness between the prediction and prototype test results under working conditions with greater discharges are unknown and should be investigated based on the prototype test data when the discharge is high enough.

## 6. Discussion

Due to the extremely complex interaction among flow, hydraulic structures, the dam foundation, and the surrounding ground, it is very difficult to accurately predict the ground vibration with existing theories and algorithms, and the errors may come from many different sources. It must be pointed out that not only an accurate simulation method for the damping ratio and Poisson’s ratio but also the influencing mechanisms of these two physical properties on the structural vibration should be further investigated to improve the prediction accuracy of ground vibration. Moreover, some errors are inevitably induced by the BP neural network prediction model because it is essentially an approximate method for establishing a nonlinear mapping, and its generalization performance can be greatly changed by artificially adjusting the fitness of the algorithm. Due to the extremely complex conditions in engineering practice, the errors can also be generated due to the vehicle vibration, background noise, and other environmental interferences in the prototype dynamic tests.

In addition, accurate treatment for the boundary conditions of the hydro-elastic model is necessary in order to further improve the experimental accuracy. The effect of the infinite ground on the vibration propagation has not been appropriately simulated by the hydro-elastic experiments, neither with nor without considering the vibration isolation system. To the authors’ knowledge, the research on the model experiment technique to appropriately simulate the infinite ground in a limited region is not reported in the published literature. The problem of an infinite ground simulation also exists in numerical analysis; thus, the viscoelastic artificial boundary, the infinite element boundary, and the free field boundary are frequently used in commercial numerical software, such as ANSYS, ABAQUS, and FLAC3D. The essence of these different boundary conditions is to dissipate vibration energy and avoid vibration reflection by adjusting the stiffness and damping of the boundary element, which can provide useful guidance for the development of the aforementioned model experiment technique. The model experiment technology to appropriately simulate the infinite ground will be further studied in following research.

Due to the space limitations, the numerous practical working conditions used in 2013, 2014, and 2015 (shown in Figure 17) are not clarified in detail. Therefore, the actual situation of the ground vibration under different working conditions is not clearly described. Most of the vibration variation laws obtained by model tests were obviously verified by the prototype experiments and some variation tendencies of the ground vibration were observed in the prototype tests but not reflected in the laboratory experiments. Specifically, the vibration amplification effect of the synchronously 5–7 m open orifice gates, the vibration reduction effect of the simultaneous discharge of the spillways and orifices in a discharge range higher than 1800 m^3^/s (especially when the orifice gates are 2–3 m open), and the vibration reduction effect of synchronously open spillway (or orifice) gates were obviously verified in the prototype observation. Moreover, the following vibration variation tendencies were found: (1) relatively high vibration intensity can be generated even in a discharge range lower than 1800 m^3^/s if the flow is not simultaneously discharged from spillways and orifices; (2) the ground vibration intensity under working conditions with flow discharged into a single stilling basin is similar to that when the same flow is discharged into both stilling basins in a discharge range of 3500 to 10,000 m^3^/s. These tendencies were not reflected in the laboratory experiments. As shown in Figure 17, the relatively high acceleration RMSs in the discharge range of 0–2500 m^3^/s are generated when the flow is discharged only from the spillways with non-uniform (even significantly different) openings of different gates. The relatively high acceleration RMSs in the discharge range of 2500–10,000 m^3^/s are mainly generated under the following conditions: (1) the flow is discharged only from spillways with large and non-uniform gate openings; (2) the flow is discharged from both spillways and orifices when the orifice gates are 4, 5, or 6 m open.

The vibrations induced by the flood discharge (or other reasons) are always there, but we can reduce the vibration intensity such that the human body cannot detect them. Therefore, the threshold for the vibration that is perceptible by the human body should be clearly clarified, and the relationship between the vibration threshold and the acceleration RMS (or amplitude) should be analyzed. According to the Standard of Vibration in Urban Area Environment (GB 10070-1988), the vibration of a residential area at night should not exceed 67 dB (i.e., 0.22 gal), which can be regarded as the perceptible vibration threshold of the human body. The vibration acceleration RMSs obtained in the prototype test were obviously smaller than this threshold, but this does not mean that the vibration cannot be felt by the human body because the acceleration RMS was much smaller than the maximum possible acceleration. If we use the peak acceleration to represent the vibration level, the vibrations generated under many working conditions are greater than the threshold. The vibration intensity is not this intense because the vibration induced by flood discharge is extremely easy to overestimate due to the susceptibility of peak acceleration and the multiple interference sources in residential areas. Moreover, the vibration amplification effect can be generated by the high-rise building structures on the surrounding ground. Thus, the difference between the vibrations of high-rise buildings and those of ground surfaces is significant, which increases the difficulty of the accurate estimation of the vibration intensity felt by the human body. All in all, the target of the optimal operation scheme is to minimize the vibration, while the impact of ground vibration on residents is appropriately estimated based on their feedback. According to that feedback, the ground vibration generated in the working conditions used in 2012 and 2013 induces physical discomfort in many residents, and the feeling of discomfort is effectively reduced by applying the optimal operation scheme used in 2014 and 2015. According to the information provided by the management department of the XHS, the maximum flow rates in the prototype working conditions used in 2016 and 2017 were approximately 6000 and 5500 m^3^/s, respectively. Based on the optimal operation scheme, the ground vibration intensities induced by flood discharge are almost always below the range of vibrations detectable by the human body.

## 7. Conclusions

In this study, an improved hydro-elastic model with a vibration isolation system was constructed according to a gravity similarity criterion and structural dynamic similarity conditions. The BP neural network prediction model was applied to establish nonlinear mapping from the model vibration to the prototype ground vibration. Based on the ground vibration prediction approach combining hydro-elastic experiments and a BP neural network, the prototype ground vibration intensities under more than 600 working conditions were predicted, and the following basic laws of vibration variation can be concluded from the results. Firstly, regardless of what the spillway gate openings are, the ground vibration will be amplified when orifice gates are synchronously 5–7 m open. Secondly, the simultaneous discharge of spillways and orifices in a discharge range higher than 1800 m^3^/s can obviously reduce the ground vibration, especially when the orifice gates are 2–3 m open. Thirdly, working conditions with synchronously and uniformly open spillway (or orifice) gates induce lower vibrations than working conditions with non-uniformly (even significantly differently) open spillways (or orifice) gates. These vibration variation laws were obviously verified in the prototype observation, which indicates that the presented hydro-elastic approach and the vibration attenuation operation scheme are effective and reasonable. A detailed optimal operation scheme was shown to reduce the ground vibration in a large discharge range of 0–30,000 m^3^/s, which has guiding significance in reducing the ground vibration induced by the flood discharge of the XHS under different working conditions. 

## Figures and Tables

**Table 1:** Comparison between the natural frequencies of the numerical model with different Poisson’s ratios.

Order	Natural Frequency		Error	Order	Natural Frequency		Error
	μ = 0.2 (0.3)	μ = 0.4			μ = 0.2 (0.3)	μ = 0.4	
1	1.9983	2.0038	0.27%	6	3.4255	3.4349	0.27%
2	2.1491	2.138	−0.52%	7	3.5154	3.5179	0.07%
3	2.9593	3.1251	5.31%	8	3.7612	3.8971	3.49%
4	3.2086	3.1917	−0.53%	9	3.8858	3.9201	0.87%
5	3.3822	3.3834	0.04%	10	4.2181	4.2649	1.10%

**Table 2:** Research on hydraulic structure modal analysis using hydro-elastic experiment technology in existing references.

Applied Methods	Research Object	The Orders Considered in Analysis	Frequency Errors between Different Methods	Mode Shape	Reference
Hydro-elastic experiment and prototype test	Bottom outlet radial gate of Three Gorges dam	First 8 orders	Errors for most orders are less than 8%.	Not considered	[36]
	Ertan arch dam	First 8 orders	Errors for most orders are less than 3%.	Not considered	[35]
Hydro-elastic experiment and numerical simulation	Water delivery valve of permanent shiplock in Three Gorges Project	First 7 orders	Less than 8.6%	Not considered	[37]
	Labyrinth weir spillway of a reservoir in Henan Province	First 3 orders	Less than 3%	Not considered	[38]
	Laxiwa arch dam	First order	Less than 1%	Corresponding mode shapes are almost the same	[39]
	Deep-hole gate of Jinpin I hydropower station	First 7 orders	Less than 5%	Not considered	[40]
	Goupitan double-curvature arch dam	First 6 orders	Less than 6%	Corresponding mode shapes are almost the same	[41,42,43]
	Xiaowan arch dam	First 3 orders (including dry and wet modes)	Less than 6% (for dry modes) and 10% (for wet modes)	Not considered	[23]
	Feilaixia overflow dam system	First 10 orders	Less than 3% (for first 4 orders) and 10% (for first 10 orders)	Not considered	[44]
	Radial gate of spillway tunnel in Baihetan hydropower station	First 3 orders	Less than 3%	Not considered	[45]
	Deep-hole radial gate of the Three Gorges Project	First 3 orders	Less than 4%	Corresponding mode shapes are almost the same	[46]

**Table 3:** Research on hydraulic structure dynamic responses using hydro-elastic experiment technology in existing references.

Applied Methods	Research Object	Tested Dynamic Response	Analysis Results	Reference
Hydro-elastic experiment and prototype test	Bottom outlet radial gate on Three Gorges dam	Peak acceleration	Errors are less than 30% (for 56% of the measuring points) and 50% (for 84% of the measuring points).	[36,47]
		Dynamic stress	Less than 8%.	
	Water delivery valve of permanent shiplock in Three Gorges Project	Dynamic displacement	Distribution laws are the same.	[37]
	Power house of Manwan hydropower station	Dynamic displacement	Maximum root mean squares (RMSs) in experiment are 24.15 μm (vertical) and 17.03 μm (horizontal). Due to the increase of reinforcement amount and concrete grade, the maximum RMSs are 10 μm (vertical) and 13 μm (horizontal) in prototype.	[48]
Hydro-elastic experiment and numerical simulation	Laxiwa arch dam	Dynamic stress	Distribution laws are the same and the errors are less than 10%.	[49]
	Xiaowan arch dam	Dynamic displacement	Variation laws are the same and the RMS errors of most measuring points are less than 10%.	[23]
	Flat gate of spillway tunnel of a hydropower station	Dynamic displacement	Less than 1%.	[50]
		Dynamic stress	Less than 15%.	
	Upper horizontal gate of a project	Acceleration	Optimal shape and operation scheme are presented and successfully applied.	[51]

**Table 4:** The optimal operation scheme aiming at reducing the ground vibration of the XHS.

Discharge(m^3^/s)	Gate Openings (m)			
	Left Stilling Basin		Right Stilling Basin	
	Spillways	Orifices	Spillways	Orifices
0→1800	0	0	0	0→2 (Gradually open as synchronously as possible)
1800→2800	0	0	0→2 (Gradually open as synchronously as possible)	2
2800→5000	0	0	2→8 (Gradually open as synchronously as possible)	2
5000→9000	0→4 (Open after the left half orifice gates are 2 m open)	0→2 (Open quickly)	8→4 (Close quickly)	2
9000→16,000	4→0 (Close quickly)	2→0 (Close quickly)	4→Fully open (Open quickly to satisfy the discharge demand)	2→3 (Open quickly)
16,000→18,000	0	0→3 (Gradually open as synchronously as possible)	Fully open	3
18,000→22,000	0→4 (Gradually open as synchronously as possible)	3	Fully open	3
22,000→30,000	4→Fully open (Gradually open as synchronously as possible)	3	Fully open	3

**Table 5:** Comparison between the ground vibration intensities under typical working conditions with similar discharges in 2012 and 2013.

Date	Upstream Water Level (m)	Discharge (m^3^/s)	Gate Opening (m)				Vertical Acceleration RMS of Point A (gal)	Reduction Ratio (%)
			Left Stilling Basin		Right Stilling Basin			
			Spillway	Orifices	Spillway	Orifices		
2012 11.6	353.50	3600		O1–O5 are approximately 4.5 m open		O6–O10 are approximately 4.5 m open	0.039	20.51
2013 7.16	371.00	3816			S8–S11 are 4.8 m open	O6–O10 are 2.9 m open	0.031	
2012 10.19	353.50	6600		O1–O5 are approximately 6.5 m open		O6–O10 are approximately 6.5 m open	0.063	55.56
2013 7.28	371.25	6767	S1 and S6 are 5.85 m open	O1–O5 are 3.5 m open	S7 and S12 are 5.85 m open	O6–O10 are 3.5 m open	0.028	

## Appendix A

A detailed description for the working conditions included in Figure 17 is given below. In the description of gate openings in Table A1, W1 indicates that the gates of the #2 and #4 orifices are 2 m open; W2 indicates that the gates of the #2, #3, and #4 orifices are 2 m open; W3 indicates that the gates of the #2 and #4 orifices are 3 m open; W4 indicates that the gates of the #2, #3 and #4 orifices are 3 m open; F and C indicate that the spillway (or orifice) gates are fully open and closed, respectively; N constants indicate that the spillway (or orifice) gates are N m open. All of the spillway (or orifice) gates corresponding to the left (or right) stilling basin have the same openings when F, C, or a constant is used to describe the gate opening.

**Table A1:** The working conditions included in Figure 17.

Case	Type	Upstream Water Level (m)	Discharge (m^3^/s)	Gate Openings (m)			
				Left Stilling Basin		Right Stilling Basin	
				Spillways	Orifices	Spillways	Orifices
1	I	380	28,843	F	2	F	2
2	I	378	25,483	F	2	F	2
3	I	376	22,649	F	2	F	2
4	I	374	19,911	F	2	F	2
5	I	372	17,311	F	2	F	2
6	I	370	15,089	F	2	F	2
7	I	370	14,313	12	2	12	2
8	I	370	13,052	10	2	10	2
9	I	370	11,488	8	2	8	2
10	I	370	9849	6	2	6	2
11	I	370	7622	4	2	4	2
12	I	370	5855	2	2	2	2
13	II	380	29,762	F	3	F	3
14	II	378	26,820	F	3	F	3
15	II	376	23,497	F	3	F	3
16	II	374	21,339	F	3	F	3
17	II	372	19,059	F	3	F	3
18	II	370	16,451	F	3	F	3
19	II	370	15,306	12	3	12	3
20	II	370	13,563	10	3	10	3
21	II	370	12,270	8	3	8	3
22	II	370	10,698	6	3	6	3
23	II	370	9078	4	3	4	3
24	II	370	6798	2	3	2	3
25	III	380	31,100	F	4	F	4
26	III	378	28,118	F	4	F	4
27	III	376	25,181	F	4	F	4
28	III	374	22,276	F	4	F	4
29	III	372	19,774	F	4	F	4
30	III	370	17,869	F	4	F	4
31	III	370	16,600	12	4	12	4
32	III	370	15,058	10	4	10	4
33	III	370	13,454	8	4	8	4
34	III	370	11,891	6	4	6	4
35	III	370	9718	4	4	4	4
36	III	370	8272	2	4	2	4
37	IV	380	14,202	C	C	F	2
38	IV	378	12,654	C	C	F	2
39	IV	376	11,118	C	C	F	2
40	IV	374	9946	C	C	F	2
41	IV	372	8664	C	C	F	2
42	IV	370	7478	C	C	F	2
43	IV	370	7155	C	C	12	2
44	IV	370	6261	C	C	10	2
45	IV	370	5631	C	C	8	2
46	IV	370	4746	C	C	6	2
47	IV	370	3797	C	C	4	2
48	IV	370	2862	C	C	2	2
49	V	380	15,109	C	C	F	3
50	V	378	13,702	C	C	F	3
51	V	376	12,270	C	C	F	3
52	V	374	11,451	C	C	F	3
53	V	372	9578	C	C	F	3
54	V	370	8281	C	C	F	3
55	V	370	8018	C	C	12	3
56	V	370	6775	C	C	10	3
57	V	370	6209	C	C	8	3
58	V	370	5304	C	C	6	3
59	V	370	4452	C	C	4	3
60	V	370	3532	C	C	2	3
61	VI	380	15,618	C	C	F	4
62	VI	378	14,172	C	C	F	4
63	VI	376	12,779	C	C	F	4
64	VI	374	11,451	C	C	F	4
65	VI	372	10,168	C	C	F	4
66	VI	370	8925	C	C	F	4
67	VI	370	8680	C	C	12	4
68	VI	370	7799	C	C	10	4
69	VI	370	6945	C	C	8	4
70	VI	370	5965	C	C	6	4
71	VI	370	5074	C	C	4	4
72	VI	370	4191	C	C	2	4
73	VII	380	15,192	C	W1	F	2
74	VII	380	15,996	C	W2	F	2
75	VII	380	16,622	C	2	F	2
76	VII	380	18,056	2	2	F	2
77	VII	380	19,421	4	2	F	2
78	VII	380	20,383	6	2	F	2
79	VII	380	21,563	8	2	F	2
80	VII	380	22,854	10	2	F	2
81	VII	380	23,632	12	2	F	2
82	VII	380	24,893	14	2	F	2
83	VII	380	25,710	16	2	F	2
84	VII	380	26,653	18	2	F	2
85	VII	380	27,661	20	2	F	2
86	VII	380	28,843	F	2	F	2
87	VIII	380	16,133	C	W3	F	3
88	VIII	380	16,686	C	W4	F	3
89	VIII	380	17,595	C	3	F	3
90	VIII	380	19,466	2	3	F	3
91	VIII	380	20,731	4	3	F	3
92	VIII	380	21,871	6	3	F	3
93	VIII	380	22,890	8	3	F	3
94	VIII	380	24,259	10	3	F	3
95	VIII	380	25,244	12	3	F	3
96	VIII	380	26,474	14	3	F	3
97	VIII	380	27,233	16	3	F	3
98	VIII	380	28,053	18	3	F	3
99	VIII	380	29,641	20	3	F	3
100	VIII	380	29,762	F	3	F	3
